# Changes in work/sleep patterns due to the COVID-19 pandemic are associated with psychological distress among Japanese workers

**DOI:** 10.3389/fpsyg.2023.1133498

**Published:** 2023-03-14

**Authors:** Tsukumi Tondokoro, Akinori Nakata, Seiichiro Tateishi, Kosuke Mafune, Mayumi Tsuji, Hajime Ando, Kiminori Odagami, Ryutaro Matsugaki, Yoshihisa Fujino

**Affiliations:** ^1^The Health Care Science Institute, Tokyo, Japan; ^2^Department of Environmental Epidemiology, Institute of Industrial Ecological Sciences, University of Occupational and Environmental Health, Japan, Kitakyushu, Japan; ^3^Graduate School of Public Health, International University of Health and Welfare, Tokyo, Japan; ^4^Disaster Occupational Health Center, Institute of Industrial Ecological Sciences, University of Occupational and Environmental Health, Japan, Kitakyushu, Japan; ^5^Department of Mental Health, Institute of Industrial Ecological Sciences, University of Occupational and Environmental Health, Japan, Kitakyushu, Japan; ^6^Department of Environmental Health, School of Medicine, University of Occupational and Environmental Health, Japan, Kitakyushu, Japan; ^7^Department of Work Systems and Health, Institute of Industrial Ecological Sciences, University of Occupational and Environmental Health, Japan, Kitakyushu, Japan; ^8^Department of Occupational Health Practice and Management, Institute of Industrial Ecological Sciences, University of Occupational and Environmental Health, Japan, Kitakyushu, Japan; ^9^Department of Preventive Medicine and Community Health, School of Medicine, University of Occupational and Environmental Health, Japan, Kitakyushu, Japan

**Keywords:** employees, financial difficulties, mental health, online survey, self-report, sleep duration, time-use, work hours

## Abstract

**Objectives:**

The coronavirus disease 2019 (COVID-19) pandemic has significantly impacted working life quality. This study investigated whether changes in work/sleep patterns due to the pandemic are related to poor psychological status among Japanese workers during the third wave of the COVID-19 pandemic (December 2020). We hypothesized that workers who experienced drastic changes in working hours and sleep duration would be at an increased risk of psychological distress.

**Methods:**

A cross-sectional self-administered Internet survey was conducted that included questions regarding socio-demographics, lifestyle, health, and occupational background and conditions. Multivariable logistic regression models were utilized to examine the association between psychological distress and a combination of changes in working hours and sleep duration.

**Results:**

Among 25,762 workers, decreased work hours and decreased sleep duration presented 2.59 times higher odds of psychological distress (95% confidence interval [CI] = 2.05–3.28) compared to those who had no changes in work hours combined with unchanged sleep duration (reference group). Increased work hours and decreased sleep duration were associated with 1.98 times higher odds of psychological distress (95% CI = 1.64–2.39).

**Conclusion:**

Our observations confirmed that decreased sleep duration could be a key factor for psychological distress, irrespective of working hours. Interestingly, workers with a combination of decreased work hours and sleep duration posed the highest risk of psychological distress. Decreased work hours accompanied by financial difficulties in the early stage of the pandemic may have caused decreased sleep duration, resulting in a high prevalence of psychological distress. Our study underlined the importance of sleep management in maintaining workers’ mental health, moreover, the need to consider situations and conditions of other daily tasks, such as work hours, for better sleep management.

## 1. Introduction

The prolonged novel coronavirus disease 2019 (COVID-19) pandemic has drastically changed our daily lives. While it has brought about some positive changes, such as less commuting and more time spent with family, it has negatively impacted mental health (Bueno-Notivol et al., 2021; Cénat et al., 2021; COVID-19 Mental Disorders Collaborators, 2021; Nochaiwong et al., 2021; OECD, 2021; Tanaka and Okamoto, 2021). Globally, due to the COVID-19 pandemic, the prevalence of mental health issues, including psychological distress, was higher than prior to the pandemic (Cénat et al., 2021; Nochaiwong et al., 2021). The estimated pooled prevalence of psychological distress among the general population was up to 50.0% in the first year of the pandemic (Cénat et al., 2021; Nochaiwong et al., 2021). Due to such psychological distress, total suicide rates have increased in Japan by 16% between July and October 2020 (Tanaka and Okamoto, 2021).

During the first year of the pandemic, many workers experienced changes in working conditions, such as forced teleworking, changes in job demands, and income reduction (financial difficulties). Moreover, workers experienced changes in their time use, especially the time used for working and sleeping.

In terms of work hours, experience of changes in work hours varies by workers. Some workers, such as health care and research laboratory workers, experienced increases in work hours during the first year of pandemic (Ischia et al., 2022; Loibner et al., 2022). According to the white paper from the Ministry of Health, Labour and Welfare, Japan (2021), workers in the information and communication industry also experienced an increase in work hours in December 2020 compared to December 2019.

In contrast, other industries, such as aviation, had a dramatic drop in demand (OECD, 2020), and the workers experienced decreases in working hours. Remote work also changed working hours; a reduction of work hours by 26 min was observed during remote work (Hallman et al., 2021), while a private survey in Japan reported that more than half of remote workers experienced longer working hours than working in the office (Japanese Trade Union Confederation, 2020). Another study also concluded that the time spent at the workstation increased by 1.5 h during a typical day for remote workers (Awada et al., 2021).

Regarding changes in sleep duration, the total sleep time increased by 1.5 h in duration at the end of the quarantine/stay-at-home condition compared to the pre-pandemic period in many countries (AMHSI Research Team et al., 2020; Raman and Coogan, 2022). Several studies on the pandemic have also indicated that remote work was associated with longer sleep duration (Hallman et al., 2021; Raman and Coogan, 2022); 34 min longer sleep duration for workdays (Hallman et al., 2021).

The Whitehall II prospective cohort study concluded that long working hours were significantly associated with shortened sleep (Virtanen et al., 2009). Furthermore, working more than 55 h per week (vs. normal work hours of 35–40 h) was related to an adjusted odds ratio (OR) of 2.25 (95% confidence interval (CI) 1.62–3.12) for short sleep of <7 h. In the same study, repeated exposure to long working hours was also associated with shortened sleep duration (OR = 2.80, 95% CI 1.20–6.53) (Virtanen et al., 2009). Given that work hours are inversely related to sleep duration, a study investigated the combined effects of working hours and sleep duration on mental health (Nakata, 2011); employees who worked more than 10 h per day with short sleep duration (<6 h/day) were at the highest risk for experiencing depressive symptoms (OR = 2.69, 95% CI 1.67–4.33) compared to those with 6–8 h of work hours and more than 6 h of sleep per day.

However, the above findings are from a pre-pandemic situation; drastic changes in our work and life conditions, including work/sleep patterns, may have been caused by COVID-19, leading to poor mental health. These past studies considered ordinal work hours and sleep duration, but not societal changes posed by the COVID-19 pandemic.

Therefore, we sought to explore how changes in work/sleep patterns due to the pandemic have impacted workers’ psychological status. This study aimed to investigate the combined association of work hours and sleep duration on psychological distress among Japanese workers during the third wave of the COVID-19 pandemic in Japan. We hypothesized that workers with increased work hours and decreased sleep duration due to the pandemic would have an increased risk of psychological distress.

## 2. Materials and methods

### 2.1. Study design and population

This Internet-based survey targeted Japanese workers, including sectional employees, managers, and executive managers as well as temporary/contract employees, self-employed, small office/home office workers, but excluding part-time workers, homemakers, students, and the unemployed. We distributed a nationwide health survey to workers registered in the research company’s panel from December 20 to 26, 2020, during the third wave of the COVID-19 pandemic in Japan (the detailed procedure is presented in our protocol paper; Fujino et al., 2021). After excluding invalid responses containing inconsistent answers, short response time (≤360 s), extremely low body weight (<30 kg), short height (<140 cm) (*n* = 6,051), and those who were already diagnosed with depression (*n* = 1,274), a total of 25,762 respondents were submitted for the final analysis.

### 2.2. Exposure measures

Participants were asked whether they had experienced changes in work hours and sleep duration due to the COVID-19 pandemic with the answering choice of increased, unchanged, or decreased. To investigate the combined association, we categorized work hours and sleep duration into nine work/sleep patterns: (i) increased work hours and increased sleep duration, (ii) increased work hours and unchanged sleep duration, (iii) increased work hours and decreased sleep duration, (iv) unchanged working hours and increased sleep duration, (v) unchanged working hours and unchanged sleep duration (reference group), (vi) unchanged work hours and decreased sleep duration, (vii) decreased work hours and increased sleep duration, (viii) decreased work hours and unchanged sleep duration, and (ix) decreased work hours and decreased sleep duration.

### 2.3. Outcome measure: Psychological distress

Psychological distress was assessed using the Japanese version of the Kessler 6 (K6) (Kessler et al., 2002), which is a short screening scale for psychological distress (Furukawa et al., 2008). K6 includes six questions regarding psychological states during the past 30 days, with five answering choices (0 = none of the time to 4 = all the time) (Furukawa et al., 2008). The scores for the six questions were then summed, and the cutoff score for mild psychological distress was set at 5 or higher, as it was suggested to be the optimal cutoff score for the Japanese population (Kessler et al., 2002; Furukawa et al., 2008; Sakurai et al., 2011).

### 2.4. Control variables

Control variables included socio-demographics (sex, age groups, marital status, educational level, living with a child or children under 12 years old [yes/no], residential area where the state of emergency was declared [yes/no], and subjective financial situation [very difficult/slightly difficult/average/comfortable/very comfortable]), lifestyle (smoking, drinking frequency, exercise frequency, time for house chores during the pandemic [increased/unchanged/decreased], and time spent with family during the pandemic [increased/unchanged/decreased]), health (body mass index, diseases currently being treated [yes/no]), and occupational background and conditions (type of industry, number of employees, one-way commuting time, teleworking preference, telework frequency, changes in job stress due to the COVID-19 pandemic [increased/unchanged/decreased], job control, and social support at work). Job control and social support at work were assessed using the job content questionnaire (Karasek et al., 1998) and divided into low/high groups by the median score of each: job control (64.0) and social support (22.0).

### 2.5. Statistical analysis

Multivariable logistic regression models were utilized to investigate the association between psychological distress and combined changes in work hours and sleep duration. We first adjusted for sex and age groups (Model 1). In the next model (Model 2), we adjusted for marital status, educational level, living with a child or children under 12 years old, residential area where the state of emergency was declared, smoking, drinking frequency, exercise frequency, time for house chores, time with family, body mass index, and diseases currently being treated, in addition to Model 1. In Model 3, we additionally adjusted for occupational background and conditions, such as type of industry, number of employees, one-way commuting time, teleworking preference, telework frequency, job control, social support, and job stress during the pandemic. Finally, in Model 4, we further adjusted for the financial situation because work hours, sleep duration, and psychological states were more likely related to the financial situation during the pandemic among workers.

In addition to the above analyses, we recorded the financial situation into two groups (difficult/not difficult) and performed a multivariate logistic regression analysis to investigate the impact of psychological distress on changes in work hours and sleep duration, in tandem with the financial situation, resulting in the creation of 18 groups.

All analyses were performed using SPSS Version 25.0 (IBM Corp., Armonk, NY, United States). The level of significance was set at *p* < 0.05.

## 3. Results

Table 1 presents the characteristics of the study participants according to their psychological state. In this study, 9,827 participants (38.1%) had mild psychological distress. A higher proportion of younger (age 20–39 years) and unmarried participants fell into the groups with psychological distress in comparison to the group without psychological distress. Nearly half of the participants with psychological distress experienced difficulty or slight difficulty in relation to their financial situation, while nearly 30% in those without psychological distress. In the group with psychological distress, 46.3% of participants experienced increased job stress and 60.0% of them experienced low social support at work. The other characteristics were similar in both groups.

**Table 1:** Characteristics of the study participants by psychological states (n = 25,762).

	*K*6 Score < 5		*K*6 Score = > 5 (Mild psychological distress)	
	*n* = 15,935		*n* = 9,827	
	Mean age: 48.5 (SD 10.3) years		Mean age: 44.6 (SD 10.5) years	
	*n*	%	*n*	%
**Sex**, male	8,861	55.6	4,271	43.5
**Age group**				
20–29 years	879	5.5	950	9.7
30–39 years	2,389	15.0	2,236	22.8
40–49 years	4,498	28.2	3,115	31.7
50–59 years	5,741	36.0	2,813	28.6
60 ≤years	2,428	15.2	713	7.3
**Marital status**				
Married	9,687	60.8	4,762	48.5
Separated/divorced	1,550	9.7	1,127	11.5
Never married	4,698	29.5	3,938	40.1
**Education**				
Junior high school	184	1.2	164	1.7
High school	4,094	25.7	2,514	25.6
Vocational school	2051	12.9	1,475	15.0
College	1,617	10.1	1,138	11.6
University	7,088	44.5	4,073	41.4
Graduate school	901	5.7	463	4.7
**Living with a child/children aged <12 years old**, yes	2,760	17.3	1,765	18.0
**Residential area**, areas where a state of emergency was declared	6,735	42.3	3,980	40.5
**Financial situation**				
Very difficult	1,225	7.7	1,812	18.4
Slightly difficult	3,953	24.8	3,370	34.3
Average	8,389	52.6	3,909	39.8
Comfortable	2,012	12.6	650	6.6
Very comfortable	356	2.2	86	0.9
**Smoking status**				
Non-smoker	8,437	52.9	5,520	56.2
Past smoker	3,374	21.2	1,821	18.5
Smoker	4,124	25.9	2,486	25.3
**Drinking frequency**				
6–7 days/week	3,611	22.7	1,848	18.8
4–5 days/week	1,274	8.0	710	7.2
2–3 days/week	1,910	12.0	1,225	12.5
<1 day/week	2,637	16.5	1,732	17.6
Almost never	6,503	40.8	4,312	43.9
**Exercise frequency**				
6–7 days/week	948	5.9	396	4.0
4–5 days/week	976	6.1	530	5.4
2–3 days/week	1,877	11.8	1,078	11.0
< 1 day/week	2,144	13.5	1,420	14.4
Almost never	9,990	62.7	6,403	65.2
**Time for house chores**				
Increased	2,183	13.7	1,790	18.2
Unchanged	13,487	84.6	7,599	77.3
Decreased	265	1.7	438	4.5
**Time with family**				
Increased	2,974	18.7	1,810	18.4
Unchanged	12,458	78.2	7,273	74.0
Decreased	503	3.2	744	7.6
**Body mass index**				
0–18.49	1,672	10.5	1,388	14.1
18.5–24.9	10,919	68.5	6,577	66.9
25 <	3,344	21.0	1,862	18.9
**Disease currently being treated**, yes	4,713	29.6	3,523	35.9
**Type of industry**				
Energy, materials, industrial machinery	602	3.8	320	3.3
Food	327	2.1	254	2.6
Beverages/tobacco products	99	0.6	35	0.4
Pharmaceuticals/medical supplies	242	1.5	141	1.4
Cosmetics/toiletries/sanitary products	83	0.5	70	0.7
Fashion and accessories	158	1.0	131	1.3
Precision machinery and office supplies	280	1.8	160	1.6
Home appliances/AV equipment	284	1.8	179	1.8
Automobiles and transportation equipment	499	3.1	366	3.7
Household goods	38	0.2	26	0.3
Hobby/sporting goods	46	0.3	28	0.3
Real estate and housing equipment	558	3.5	333	3.4
Information and communication	833	5.2	427	4.3
Distribution and retail	1,087	6.8	639	6.5
Finance/insurance	734	4.6	436	4.4
Transportation and leisure	408	2.6	226	2.3
Restaurant and other services	761	4.8	508	5.2
Public offices and organizations	1,156	7.3	609	6.2
Education, medical services, religion	2,574	16.2	1,717	17.5
Newspapers, magazines, television, radio, advertising, and other mass media	122	0.8	87	0.9
Market research	30	0.2	15	0.2
Other	5,014	31.5	3,120	31.7
**Number of employees**				
1 (Freelance)	1,599	10.0	798	8.1
2–4	1,255	7.9	678	6.9
5–9	992	6.2	582	5.9
10–29	1,624	10.2	1,063	10.8
30–49	891	5.6	631	6.4
50–99	1,448	9.1	986	10.0
100–499	2,940	18.4	1,992	20.3
500–999	1,138	7.1	759	7.7
> 1,000	2,767	17.4	1,682	17.1
> 10,000	1,281	8.0	656	6.7
**Commuting time**				
> 2 h	306	1.9	245	2.5
1–2 h(s)	2,075	13.0	1,398	14.2
30 min-1 h	4,267	26.8	2,831	28.8
< 30 min	6,781	42.6	3,932	40.0
Almost never	2,506	15.7	1,421	14.5
**Telework preference**				
Willing to work from home	4,328	27.2	3,714	37.8
Neither	4,823	30.3	2,770	28.2
Willing to work at office	6,784	42.6	3,343	34.0
**Telework frequency**				
> 4 days/week	1,672	10.5	920	9.4
> 2 days/week	888	5.6	503	5.1
> 1 day/week	529	3.3	306	3.1
> 1 day/month	355	2.2	239	2.4
Almost never	12,491	78.4	7,859	80.0
**Job stress**				
Increased	2,922	18.3	4,551	46.3
Unchanged	12,399	77.8	4,955	50.4
Decreased	614	3.9	321	3.3
**Social support**				
Low	6,630	41.6	5,893	60.0
High	9,305	58.4	3,934	40.0
**Job control**				
Low	7,778	48.8	5,885	59.9
High	8,157	51.2	3,942	40.1

Table 2 highlights the characteristics of the study participants by nine groups of changes in work/sleep patterns due to the pandemic. In the group with decreased working hours and decreased sleep duration, more than 30% of participants had experienced extreme difficulty in relation to their financial situation. In the group with increased working hours and decreased sleep duration, 81.6% experienced increases in job stress due to the pandemic.

**Table 2:** Characteristics of the study participants by changes in work/sleep patterns.

Work hours	Increased						Unchanged						Decreased					
Sleep duration	Increasedn = 347 Mean age: 42.5 (SD 11.3) years		Unchanged*n* = 1,049 Mean age: 44.6 (SD 10.2) years		Decreased*n* = 692 Mean age: 45.1 (SD 10.1) years		Increased*n* = 1,202 Mean age: 43.5 (SD 11.0) years		Unchanged*n* = 18,185 Mean age: 47.6 (SD 10.5) years		Decreased*n* = 1,624 Mean age: 45.8 (SD 10.5) years		Increased*n* = 601 Mean age: 43.7 (SD 11.1) years		Unchanged*n* = 1,625 Mean age: 48.4 (SD 10.1) years		Decreasedn = 437 Mean age: 46.6 (SD 10.8) years	
	*n*	%	*n*	%	*n*	%	*n*	%	*n*	%	*n*	%	*n*	%	*n*	%	*n*	%
**Sex**, male	140	40.3	486	46.3	312	45.1	425	35.4	9,795	53.9	648	39.9	229	38.1	892	54.9	205	46.9
**Age group**																		
20–29 years	55	15.9	95	9.1	64	9.2	150	12.5	1,140	6.3	127	7.8	79	13.1	81	5.0	38	8.7
30–39 years	93	26.8	223	21.3	129	18.6	314	26.1	3,043	16.7	327	20.1	148	24.6	268	16.5	80	18.3
40–49 years	85	24.5	357	34.0	231	33.4	349	29.0	5,317	29.2	533	32.8	173	28.8	438	27.0	130	29.7
50–59 years	92	26.5	309	29.5	232	33.5	294	24.5	6,246	34.3	487	30.0	143	23.8	610	37.5	141	32.3
60 = < years	22	6.3	65	6.2	36	5.2	95	7.9	2,439	13.4	150	9.2	58	9.7	228	14.0	48	11.0
**Marital status**																		
Married	179	51.6	582	55.5	365	52.7	606	50.4	10,499	57.7	861	53.0	270	44.9	878	54.0	209	47.8
Separated/divorced	27	7.8	104	9.9	69	10.0	119	9.9	1,854	10.2	232	14.3	50	8.3	170	10.5	52	11.9
Never married	141	40.6	363	34.6	258	37.3	477	39.7	5,832	32.1	531	32.7	281	46.8	577	35.5	176	40.3
**Education**																		
Junior high school	4	1.2	11	1.0	15	2.2	10	0.8	253	1.4	16	1.0	6	1.0	23	1.4	10	2.3
High school	46	13.3	192	18.3	162	23.4	251	20.9	4,847	26.7	463	28.5	116	19.3	418	25.7	113	25.9
Vocational school	31	8.9	136	13.0	100	14.5	156	13.0	2,478	13.6	264	16.3	72	12.0	223	13.7	66	15.1
College	25	7.2	115	11.0	78	11.3	140	11.6	1,889	10.4	211	13.0	65	10.8	173	10.6	59	13.5
University	208	59.9	501	47.8	289	41.8	567	47.2	7,802	42.9	608	37.4	312	51.9	709	43.6	165	37.8
Graduate school	33	9.5	94	9.0	48	6.9	78	6.5	916	5.0	62	3.8	30	5.0	79	4.9	24	5.5
**Living with a child/children aged <12 years old**, yes	66	19.0	212	20.2	140	20.2	201	16.7	3,151	17.3	356	21.9	84	14.0	235	14.5	80	18.3
**Residential area**, areas where a state of emergency was declared	209	60.2	487	46.4	301	43.5	645	53.7	7,123	39.2	640	39.4	363	60.4	737	45.4	210	48.1
**Financial situation**																		
Very difficult	43	12.4	107	10.2	150	21.7	101	8.4	1,847	10.2	308	19.0	79	13.1	268	16.5	134	30.7
Slightly difficult	85	24.5	283	27.0	209	30.2	331	27.5	4,985	27.4	582	35.8	177	29.5	533	32.8	138	31.6
Average	147	42.4	501	47.8	259	37.4	576	47.9	9,180	50.5	626	38.5	245	40.8	637	39.2	127	29.1
Comfortable	59	17.0	145	13.8	68	9.8	155	12.9	1,858	10.2	93	5.7	89	14.8	162	10.0	33	7.6
Very comfortable	13	3.7	13	1.2	6	0.9	39	3.2	315	1.7	15	0.9	11	1.8	25	1.5	5	1.1
**Smoking status**																		
Non-smoker	213	61.4	603	57.5	394	56.9	729	60.6	9,759	53.7	908	55.9	341	56.7	796	49.0	214	49.0
Past smoker	57	16.4	190	18.1	121	17.5	222	18.5	3,729	20.5	292	18.0	125	20.8	376	23.1	83	19.0
Smoker	77	22.2	256	24.4	177	25.6	251	20.9	4,697	25.8	424	26.1	135	22.5	453	27.9	140	32.0
**Drinking frequency**																		
6–7 days/week	69	19.9	203	19.4	101	14.6	196	16.3	4,011	22.1	277	17.1	119	19.8	392	24.1	91	20.8
4–5 days/week	37	10.7	84	8.0	45	6.5	99	8.2	1,383	7.6	118	7.3	59	9.8	128	7.9	31	7.1
2–3 days/week	51	14.7	128	12.2	84	12.1	164	13.6	2,193	12.1	186	11.5	80	13.3	205	12.6	44	10.1
< 1 day/week	69	19.9	203	19.4	126	18.2	233	19.4	2,958	16.3	286	17.6	125	20.8	300	18.5	69	15.8
Almost never	121	34.9	431	41.1	336	48.6	510	42.4	7,640	42.0	757	46.6	218	36.3	600	36.9	202	46.2
**Exercise frequency**																		
6–7 days/week	36	10.4	50	4.8	31	4.5	83	6.9	938	5.2	70	4.3	33	5.5	87	5.4	16	3.7
4–5 days/week	29	8.4	66	6.3	32	4.6	86	7.2	1,039	5.7	84	5.2	41	6.8	105	6.5	24	5.5
2–3 days/week	74	21.3	132	12.6	69	10.0	169	14.1	2,013	11.1	160	9.9	87	14.5	204	12.6	47	10.8
< 1 day/week	46	13.3	160	15.3	117	16.9	198	16.5	2,401	13.2	219	13.5	115	19.1	241	14.8	67	15.3
Almost never	162	46.7	641	61.1	443	64.0	666	55.4	11,794	64.9	1,091	67.2	325	54.1	988	60.8	283	64.8
**Time for house chores**																		
Increased	206	59.4	256	24.4	181	26.2	484	40.3	1,574	8.7	407	25.1	331	55.1	387	23.8	147	33.6
Unchanged	126	36.3	744	70.9	345	49.9	686	57.1	16,474	90.6	1,086	66.9	248	41.3	1,194	73.5	183	41.9
Decreased	15	4.3	49	4.7	166	24.0	32	2.7	137	0.8	131	8.1	22	3.7	44	2.7	107	24.5
**Time with family**																		
Increased	205	59.1	294	28.0	142	20.5	530	44.1	2,282	12.5	361	22.2	329	54.7	523	32.2	118	27.0
Unchanged	111	32.0	668	63.7	371	53.6	592	49.3	15,513	85.3	1,098	67.6	216	35.9	985	60.6	177	40.5
Decreased	31	8.9	87	8.3	179	25.9	80	6.7	390	2.1	165	10.2	56	9.3	117	7.2	142	32.5
**Body mass index**																		
0–18.49	40	11.5	128	12.2	95	13.7	164	13.6	2,091	11.5	212	13.1	55	9.2	208	12.8	67	15.3
18.5–24.9	235	67.7	713	68.0	452	65.3	817	68.0	12,450	68.5	1,021	62.9	448	74.5	1,078	66.3	282	64.5
25 <	72	20.7	208	19.8	145	21.0	221	18.4	3,644	20.0	391	24.1	98	16.3	339	20.9	88	20.1
**Disease currently being treated**, yes	108	31.1	361	34.4	285	41.2	369	30.7	5,579	30.7	627	38.6	185	30.8	541	33.3	181	41.4
**Type of industry**																		
Energy, materials, industrial machinery	15	4.3	30	2.9	18	2.6	32	2.7	658	3.6	60	3.7	15	2.5	72	4.4	22	5.0
Food	4	1.2	23	2.2	17	2.5	17	1.4	394	2.2	41	2.5	18	3.0	49	3.0	18	4.1
Beverages/tobacco products	2	0.6	7	0.7	1	0.1	7	0.6	101	0.6	5	0.3	3	0.5	6	0.4	2	0.5
Pharmaceuticals/medical supplies	6	1.7	16	1.5	13	1.9	24	2.0	264	1.5	25	1.5	8	1.3	23	1.4	4	0.9
Cosmetics/toiletries/sanitary products	4	1.2	6	0.6	8	1.2	10	0.8	96	0.5	8	0.5	5	0.8	10	0.6	6	1.4
Fashion and accessories	3	0.9	12	1.1	8	1.2	20	1.7	173	1.0	16	1.0	13	2.2	36	2.2	8	1.8
Precision machinery and office supplies	8	2.3	20	1.9	7	1.0	21	1.7	314	1.7	20	1.2	6	1.0	35	2.2	9	2.1
Home appliances/AV equipment	10	2.9	24	2.3	10	1.4	24	2.0	329	1.8	19	1.2	8	1.3	29	1.8	10	2.3
Automobiles and transportation equipment	16	4.6	31	3.0	19	2.7	38	3.2	554	3.0	59	3.6	21	3.5	107	6.6	20	4.6
Household goods	0	0.0	4	0.4	2	0.3	3	0.2	44	0.2	6	0.4	0	0.0	4	0.2	1	0.2
Hobby/sporting goods	4	1.2	2	0.2	0	0.0	3	0.2	56	0.3	2	0.1	1	0.2	3	0.2	3	0.7
Real estate and housing equipment	2	0.6	28	2.7	23	3.3	36	3.0	662	3.6	56	3.4	11	1.8	56	3.4	17	3.9
Information and communication	39	11.2	71	6.8	34	4.9	105	8.7	823	4.5	71	4.4	40	6.7	64	3.9	13	3.0
Distribution and retail	27	7.8	55	5.2	40	5.8	85	7.1	1,258	6.9	110	6.8	40	6.7	90	5.5	21	4.8
Finance/insurance	19	5.5	53	5.1	36	5.2	69	5.7	808	4.4	74	4.6	25	4.2	66	4.1	20	4.6
Transportation and leisure	14	4.0	17	1.6	12	1.7	38	3.2	380	2.1	35	2.2	52	8.7	71	4.4	15	3.4
Restaurant and other services	12	3.5	45	4.3	32	4.6	46	3.8	827	4.5	80	4.9	63	10.5	133	8.2	31	7.1
Public offices and organizations	17	4.9	79	7.5	50	7.2	66	5.5	1,364	7.5	88	5.4	29	4.8	59	3.6	13	3.0
Education, medical services, religion	54	15.6	235	22.4	173	25.0	202	16.8	3,039	16.7	332	20.4	44	7.3	161	9.9	51	11.7
Newspapers, magazines, television, radio, advertising, and other mass media	6	1.7	12	1.1	8	1.2	12	1.0	126	0.7	11	0.7	7	1.2	15	0.9	12	2.7
Market research	0	0.0	3	0.3	0	0.0	0	0.0	38	0.2	2	0.1	0	0.0	2	0.1	0	0.0
Other	85	24.5	276	26.3	181	26.2	344	28.6	5,877	32.3	504	31.0	192	31.9	534	32.9	141	32.3
**Number of employees**																		
1 (Freelance)	37	10.7	82	7.8	41	5.9	95	7.9	1,648	9.1	110	6.8	74	12.3	254	15.6	56	12.8
2–4	8	2.3	50	4.8	47	6.8	71	5.9	1,450	8.0	108	6.7	35	5.8	134	8.2	30	6.9
5–9	16	4.6	38	3.6	41	5.9	71	5.9	1,164	6.4	97	6.0	33	5.5	90	5.5	24	5.5
10–29	21	6.1	82	7.8	74	10.7	114	9.5	1,991	10.9	174	10.7	53	8.8	141	8.7	37	8.5
30–49	16	4.6	53	5.1	42	6.1	67	5.6	1,097	6.0	112	6.9	26	4.3	86	5.3	23	5.3
50–99	20	5.8	89	8.5	56	8.1	109	9.1	1,746	9.6	182	11.2	55	9.2	140	8.6	37	8.5
100–499	64	18.4	196	18.7	132	19.1	215	17.9	3,510	19.3	347	21.4	95	15.8	287	17.7	86	19.7
500–999	42	12.1	105	10.0	72	10.4	112	9.3	1,259	6.9	114	7.0	46	7.7	104	6.4	43	9.8
> 1,000	73	21.0	234	22.3	132	19.1	221	18.4	3,071	16.9	287	17.7	123	20.5	242	14.9	66	15.1
> 10,000	50	14.4	120	11.4	55	7.9	127	10.6	1,249	6.9	93	5.7	61	10.1	147	9.0	35	8.0
**Commuting time**																		
> 2 h	34	9.8	31	3.0	28	4.0	32	2.7	337	1.9	23	1.4	22	3.7	29	1.8	15	3.4
1–2 h(s)	78	22.5	172	16.4	119	17.2	212	17.6	2,257	12.4	247	15.2	89	14.8	243	15.0	56	12.8
30 min-1 h	87	25.1	321	30.6	193	27.9	339	28.2	4,895	26.9	476	29.3	191	31.8	472	29.0	124	28.4
< 30 min	84	24.2	386	36.8	281	40.6	416	34.6	7,889	43.4	670	41.3	192	31.9	643	39.6	152	34.8
Almost never	64	18.4	139	13.3	71	10.3	203	16.9	2,807	15.4	208	12.8	107	17.8	238	14.6	90	20.6
**Telework preference**																		
Willing to work from home	213	61.4	462	44.0	266	38.4	560	46.6	5,009	27.5	545	33.6	273	45.4	548	33.7	166	38.0
Neither	59	17.0	255	24.3	175	25.3	287	23.9	5,656	31.1	474	29.2	147	24.5	434	26.7	106	24.3
Willing to work at office	75	21.6	332	31.6	251	36.3	355	29.5	7,520	41.4	605	37.3	181	30.1	643	39.6	165	37.8
**Telework frequency**																		
> 4 days/week	117	33.7	161	15.3	65	9.4	234	19.5	1,551	8.5	102	6.3	124	20.6	193	11.9	45	10.3
> 2 days/week	55	15.9	123	11.7	38	5.5	142	11.8	748	4.1	47	2.9	65	10.8	127	7.8	46	10.5
> 1 day/week	24	6.9	63	6.0	36	5.2	48	4.0	486	2.7	40	2.5	34	5.7	84	5.2	20	4.6
> 1 day/month	11	3.2	41	3.9	19	2.7	20	1.7	375	2.1	38	2.3	23	3.8	50	3.1	17	3.9
Almost never	140	40.3	661	63.0	534	77.2	758	63.1	15,025	82.6	1,397	86.0	355	59.1	1,171	72.1	309	70.7
**Job stress**																		
Increased	191	55.0	584	55.7	565	81.6	361	30.0	3,732	20.5	1,010	62.2	193	32.1	587	36.1	250	57.2
Unchanged	117	33.7	423	40.3	110	15.9	700	58.2	14,182	78.0	581	35.8	242	40.3	885	54.5	114	26.1
Decreased	39	11.2	42	4.0	17	2.5	141	11.7	271	1.5	33	2.0	166	27.6	153	9.4	73	16.7
**Social support**																		
Low	171	49.3	556	53.0	439	63.4	506	42.1	8,618	47.4	943	58.1	270	44.9	783	48.2	237	54.2
High	176	50.7	493	47.0	253	36.6	696	57.9	9,567	52.6	681	41.9	331	55.1	842	51.8	200	45.8
**Job control**																		
Low	184	53.0	525	50.0	345	49.9	602	50.1	9,715	53.4	959	59.1	294	48.9	810	49.8	229	52.4
High	163	47.0	524	50.0	347	50.1	600	49.9	8,470	46.6	665	40.9	307	51.1	815	50.2	208	47.6

Independent associations between psychological distress and work hours and sleep duration, respectively, as estimated by stepwise logistic regression models, are denoted in Table 3. In Model 4, participants with increased working hours had significantly higher odds of experiencing psychological distress (odds ratio [OR] = 1.15, 95% confidence interval [CI] = 1.03–1.28) compared to those with unchanged work hours, while decreased work hours showed an insignificant association. Regarding sleep duration, those who experienced decreased sleep duration showed OR of 1.97 (95% CI = 1.79–2.18) compared to unchanged sleep duration.

**Table 3:** Independent association between psychological distress and changes in work hours and sleep duration, respectively.

	*n*	Crude OR	95% CI	Model 1		Model 2		Model 3		Model 4	
	Case/Exposed			OR	95% CI	OR	95% CI	OR	95% CI	OR	95% CI
Work hours											
Increased	1163/2088	2.26^c^	2.06–2.47	2.10^c^	1.92–2.30	1.56^c^	1.41–1.72	1.12^a^	1.01–1.25	1.15^a^	1.03–1.28
Unchanged	7522/21011	Reference		Reference		Reference		Reference		Reference	
Decreased	1142/2663	1.35^c^	1.24–1.46	1.35^c^	1.24–1.46	1.15^b^	1.05–1.26	1.05	0.96–1.16	0.99	0.90–1.09
Sleep duration											
Increased	871/2150	1.31^c^	1.20–1.43	1.14	1.04–1.25	1.10	1.00–1.22	1.05	0.94–1.17	1.06	0.95–1.18
Unchanged	7139/20859	Reference		Reference		Reference		Reference		Reference	
Decreased	1817/2753	3.73^c^	3.43–4.06	3.59^c^	3.30–3.91	3.14^c^	2.86–3.43	2.11^c^	1.91–2.32	1.97^c^	1.79–2.18

Model 1: adjusted for sex and age group. Model 2: Model 1 + adjusted for marital status, educational level, living with a child/children under 12 years old, residential area, smoking, drinking, exercise, time for house chores (increased/unchanged/decreased), time with family (increased/unchanged/decreased), body mass index, disease currently being treated (yes/no), and sleep duration for analysis for work hours. Model 3: Model 2 + adjusted for type of industry, number of employees, commuting time, teleworking preference, telework frequency, job control, social support, and job stress, and work hours for analysis of sleep duration. Model 4: Model 3 + financial situation. ^a^*p* < 0.05, ^b^*p* < 0.01, ^c^*p* < 0.001.

Table 4 presents associations between psychological distress and changes in work/sleep combined patterns by stepwise logistic regression models. Notably, participants had more risk of experiencing psychological distress when sleep duration decreased. Those who reported decreases in work and sleep duration presented the highest odds of experiencing psychological distress (OR = 2.59, 95% CI = 2.05–3.28) among all other groups. This group had a drop of OR from 2.93 in Model 3 to 2.59 in Model 4, which was additionally adjusted for the financial situation. Those who experienced increased work hours and decreased sleep duration had higher odds of experiencing psychological distress (OR = 1.98, 95% CI = 1.64–2.39) compared to those who reported no changes in work hours and sleep duration (reference group).

**Table 4:** Association between psychological distress and changes in work/sleep patterns.

Work hours	Sleep duration	*n*	Crude OR	95% CI	Model 1		Model 2		Model 3		Model 4	
		Case/Exposed			OR	95% CI	OR	95% CI	OR	95% CI	OR	95% CI
Increased	Increased	169/347	1.92^c^	1.55–2.37	1.66^c^	1.34–2.06	1.71^c^	1.37–2.14	1.21	0.95–1.53	1.21	0.95–1.54
	Unchanged	512/1049	1.92^c^	1.70–2.18	1.77^c^	1.56–2.01	1.73^c^	1.52–1.97	1.21^b^	1.06–1.39	1.24^b^	1.08–1.43
	Decreased	482/692	4.63^c^	3.93–5.46	4.39^c^	3.72–5.19	3.73^c^	3.13–4.45	2.05^c^	1.70–2.47	1.98^c^	1.64–2.39
Unchanged	Increased	467/1202	1.28^c^	1.14–1.45	1.11	0.98–1.25	1.11	0.97–1.26	1.08	0.94–1.23	1.08	0.94–1.24
	Unchanged	6028/18185	Reference		Reference		Reference		Reference		Reference	
	Decreased	1027/1624	3.47^c^	3.12–3.86	3.31^c^	2.98–3.69	3.09^c^	2.77–3.45	2.08^c^	1.85–2.34	1.94^c^	1.72–2.18
Decreased	Increased	235/601	1.30^b^	1.10–1.53	1.14	0.96–1.35	1.10	0.92–1.31	1.05	0.87–1.27	1.01	0.83–1.22
	Unchanged	599/1625	1.18^b^	1.06–1.31	1.21^c^	1.09–1.35	1.18^b^	1.05–1.31	1.01	0.90–1.13	0.94	0.83–1.06
	Decreased	308/437	4.82^c^	3.91–5.93	4.82^c^	3.90–5.95	3.87^c^	3.11–4.82	2.93^c^	2.32–3.69	2.59^c^	2.05–3.28

Model 1: adjusted for sex and age group. Model 2: Model 1 + adjusted for marital status, educational level, living with a child/children under 12 years old (yes/no), residential area, smoking, drinking frequency, exercise frequency, time for house chores (increased/unchanged/decreased), time with family (increased/unchanged/decreased), body mass index, and disease currently being treated (yes/no). Model 3: Model 2 + adjusted for type of industry, number of employees, commuting time, teleworking preference, telework frequency, job control, social support, and job stress (increased/unchanged/decreased). Model 4: Model 3 + financial situation. ^a^*p* < 0.05. ^b^*p* < 0.01. ^c^*p* < 0.001.

Additionally, our supplementary analysis (Supplementary Table S1) indicated that those who experienced decreased work hours and decreased sleep duration coupled with financial difficulties had an OR of 5.88 (95% CI = 4.29–8.06) compared to those who experienced unchanged work hours and unchanged sleep duration coupled with no financial difficulties (reference group), which was the highest among all other groups.

## 4. Discussion

This study investigated the association between changes in work/sleep patterns and psychological distress among Japanese workers during the COVID-19 pandemic. To our knowledge, this is the first study that specifically examined the combined effect of changes in work hours and sleep duration from pre-pandemic to in-pandemic periods on psychological distress. This study primarily questioned whether changes in work hours and sleep duration would pose a higher risk of psychological distress compared to when the work hours and sleep duration remained unchanged. We found an increased risk of psychological distress among those who had decreased sleep duration, irrespective of changes in work hours. Notably, workers with decreased work hours and decreased sleep duration had the highest odds of psychological distress (OR = 2.59, 95% CI = 2.05–3.28), followed by workers with increased work hours and decreased sleep duration (OR = 1.98, 95% CI = 1.64–2.39). This finding outlines that reduced sleep duration is a major factor related to increased psychological distress.

Moreover, we found that the risk of psychological distress was the highest among those with a combination of decreased work hours and sleep duration in the early stages of the pandemic, which was contrary to our expectations. A possible key factor that led to this result was financial impact of the pandemic on this decreased work hours and decreased sleep duration group. We found that more than 30% of participants in this group reported experiencing extreme difficulty in relation to their financial situation (Table 2). This group also experienced a drop of OR from 2.93 to 2.59 when adjusted for the financial situation in the final model in Table 4. Additionally, in our sub-analysis, we found a higher risk of psychological distress (OR = 5.88, 95% CI = 4.29–8.06) among those with decreased work hours and decreased sleep duration coupled with financial difficulties compared to unchanged work hours and unchanged sleep duration coupled with no financial difficulties (Supplementary Table S1). Previous studies have also supported our hypothesis; people experiencing financial difficulties had higher rates of psychological distress during the COVID-19 crisis (Griffiths et al., 2021; Nagasu et al., 2021; OECD, 2021; Ettman et al., 2022; Sekścińska et al., 2022).

The increased risk of psychological distress, with increased work hours and decreased sleep duration, was in line with a past cross-sectional study in an ordinal pre-pandemic work situation (Nakata, 2011), which presented longer work hours of 10+ h per day with short sleep duration (<6 h/day) to have 2.69 times higher odds of having depressive symptoms compared to normal working hours (6–8 h/day with more than 6 h/day of sleep).

In the early stages of the pandemic, many workers experienced unstable working conditions, such as decreased quantitative job demands, decreased work hours, and reduced income. Despite this situation, the provision of job-related services and support (e.g., in-person job consultations and career seminars) was limited to reduced human contact, resulting in workers having poorer access to job-related services and support. According to statistics from the Ministry of Health, Labour and Welfare, Japan, the number of new job applications in public employment service centers in Japan dropped by approximately 10,000 from 2019 to 2020 (Ministry of Health Labour and Welfare, Japan, 2022). Such unstable working conditions and the limited provision of job-related services and support during the pandemic are more likely to lead to financial difficulties for several workers.

A previous occupational cohort study revealed strong associations between financial difficulties and sleep problems such as shortened sleep duration (Lallukka et al., 2012). In addition to financial difficulties and decreased sleep duration, uncertainty over the prolonged pandemic may have increased psychological distress among workers. Moreover, fewer socializing opportunities, limited access to mental health services and support, and fewer opportunities to seek help during the pandemic (Campion et al., 2020; Moreno et al., 2020) might have heightened the risk of psychological distress.

The strengths of this study are as follows. First, we assessed changes in work/sleep patterns among Japanese workers from pre-pandemic to in-pandemic periods, unlike previous studies that cross-sectionally assessed work/sleep patterns on typical workdays. Second, we employed self-reports to evaluate sudden changes in work/sleep patterns due to the pandemic. Most previous studies objectively assessed changes in working hours and sleep duration by the number of hours. However, evaluating the sudden changes caused by COVID-19 with numbers by self-report would lead to potential biases, that is, a recall bias in the number of hours before the pandemic. Additionally, sufficient working hours and sleep duration vary among individuals. Third, we included both changes in work hours and sleep duration due to the COVID-19 pandemic to examine the acute impact on psychological distress. Combining changes in work hours and sleep duration would reflect the time use of workers because they spend a large amount of time working and sleeping during the day.

However, this study had several limitations. First, although many workers experienced increased sleep duration in the early stages of the pandemic, our study focused on workers with decreased sleep duration. Second, this study utilizes an online closed survey, meaning that participants were recruited from the survey company’s panel, which may have led to selection bias. Third, we were unable to confirm the participants’ pre-pandemic psychological state. They may have already developed psychological distress prior to the pandemic. Fourth, we were unable to identify whether participants experienced decreased sleep duration because of the stressful situation from the pandemic, or whether they simply could not set aside time for sleep because they had other tasks to do. Finally, there might be other co-factors, such as napping, side jobs, and time-use on free days, which may have impacted psychological distress among Japanese workers.

## 5. Conclusion

This study confirmed that decreased sleep duration could be a key factor in psychological distress, irrespective of working hours. Moreover, contrary to our expectations, workers with a combination of decreased work hours and sleep duration posed the highest risk of psychological distress. Decreased work hours accompanied by financial difficulties in the early stage of the pandemic may have caused decreased sleep duration, resulting in a high prevalence of psychological distress. Our study underlined the importance of sleep management in maintaining workers’ mental health as well as the need to consider situations and conditions of other daily tasks, such as work hours, for better sleep management.

## Data availability statement

The datasets presented in this article are not readily available because it is not open to the public. Requests to access the datasets should be directed to YF, zenq@med.uoeh-u.ac.jp.

## Ethics statement

The studies involving human participants were reviewed and approved by the Ethics Committee of the University of Occupational and Environmental Health, Japan and the Ethics Committee of the International University of Health and Welfare. The patients/participants provided their written informed consent to participate in this study.

## Author contributions

TT contributed to data analysis and writing. YF, ST, KM, MT, HA, KO, and RM contributed to the research design and data collection and provided useful suggestions during the analysis. AN supervised the analysis and drafting of the manuscript and reviewed the manuscript for intellectual content. All authors have contributed to the manuscript and approved the submitted version.

## Funding

This study was supported and partly funded by a research grant from the University of Occupational and Environmental Health, Japan; the Japanese Ministry of Health, Labour and Welfare (H30-josei-ippan-002, H30-roudou-ippan-007, 19JA1004, 20JA1006, 210301-1, 19H01763, and 20HB1004); Anshin Zaidan; the Collabo-Health Study Group; Hitachi Systems, Ltd.; scholarship donations from Chugai Pharmaceutical Co., Ltd.; and The Health Care Science Institute. The funder was not involved in the study design, collection, analysis, data interpretation, writing of this article, or the decision to submit it for publication.

## Conflict of interest

The authors declare that the research was conducted in the absence of any commercial or financial relationships that could be construed as a potential conflict of interest.

## Publisher’s note

All claims expressed in this article are solely those of the authors and do not necessarily represent those of their affiliated organizations, or those of the publisher, the editors and the reviewers. Any product that may be evaluated in this article, or claim that may be made by its manufacturer, is not guaranteed or endorsed by the publisher.
