# Supplementary material for: Changes in work/sleep patterns due to the COVID-19 pandemic are associated with psychological distress among Japanese workers
Source: Front Psychol. 2023 Mar 14;14:1133498. doi: 10.3389/fpsyg.2023.1133498 (PMC10043417; doi:10.3389/fpsyg.2023.1133498)
Supplement: Supplementary file 1 [file Table_1.docx]

| Supplementary Table S1. Association between psychological distress and changes in work/sleep patterns with financial situation | | | | | | | | | | | | | | | | | | | | | | | | | | |
| --- | --- | --- | --- | --- | --- | --- | --- | --- | --- | --- | --- | --- | --- | --- | --- | --- | --- | --- | --- | --- | --- | --- | --- | --- | --- | --- |
|  |  |  | n |  |  |  |  |  |  | Model 1 | |  |  |  |  | Model 2 | |  |  |  |  | Model 3 | |  |  |  |
| **Financial situation** | **Work hours** | **Sleep duration** | Case/ Exposed | Crude OR |  | 95% CI | | |  | OR |  | 95% CI | | |  | OR |  | 95% CI | | |  | OR |  | 95% CI | | |
| Difficult | increased | increased | 83/128 | 5.16 | ^c^ | 3.58 | - | 7.43 |  | 4.73 | ^c^ | 3.26 | - | 6.86 |  | 4.63 | ^c^ | 3.17 | - | 6.76 |  | 2.91 | ^c^ | 1.96 | - | 4.31 |
|  |  | unchanged | 233/390 | 4.15 | ^c^ | 3.38 | - | 5.10 |  | 4.03 | ^c^ | 3.26 | - | 4.97 |  | 3.77 | ^c^ | 3.05 | - | 4.67 |  | 2.34 | ^c^ | 1.87 | - | 2.93 |
|  |  | decreased | 284/359 | 10.59 | ^c^ | 8.18 | - | 13.71 |  | 10.38 | ^c^ | 8.00 | - | 13.47 |  | 8.56 | ^c^ | 6.55 | - | 11.18 |  | 4.32 | ^c^ | 3.27 | - | 5.71 |
|  | unchanged | increased | 217/432 | 2.82 | ^c^ | 2.33 | - | 3.43 |  | 2.49 | ^c^ | 2.05 | - | 3.04 |  | 2.39 | ^c^ | 1.96 | - | 2.93 |  | 2.05 | ^c^ | 1.66 | - | 2.53 |
|  |  | unchanged | 3038/6832 | 2.24 | ^c^ | 2.10 | - | 2.39 |  | 2.35 | ^c^ | 2.21 | - | 2.51 |  | 2.29 | ^c^ | 2.14 | - | 2.45 |  | 2.04 | ^c^ | 1.90 | - | 2.18 |
|  |  | decreased | 615/890 | 6.26 | ^c^ | 5.39 | - | 7.25 |  | 6.23 | ^c^ | 5.36 | - | 7.25 |  | 5.74 | ^c^ | 4.92 | - | 6.70 |  | 3.53 | ^c^ | 3.00 | - | 4.15 |
|  | decreased | increased | 126/256 | 2.71 | ^c^ | 2.11 | - | 3.48 |  | 2.42 | ^c^ | 1.88 | - | 3.12 |  | 2.27 | ^c^ | 1.75 | - | 2.94 |  | 1.97 | ^c^ | 1.50 | - | 2.59 |
|  |  | unchanged | 372/801 | 2.43 | ^c^ | 2.10 | - | 2.80 |  | 2.60 | ^c^ | 2.24 | - | 3.01 |  | 2.45 | ^c^ | 2.11 | - | 2.85 |  | 1.93 | ^c^ | 1.64 | - | 2.26 |
|  |  | decreased | 214/272 | 10.32 | ^c^ | 7.70 | - | 13.84 |  | 10.62 | ^c^ | 7.89 | - | 14.29 |  | 8.42 | ^c^ | 6.21 | - | 11.40 |  | 5.88 | ^c^ | 4.29 | - | 8.06 |
| Not  difficult | increased | increased | 86/219 | 1.81 | ^c^ | 1.38 | - | 2.38 |  | 1.54 | ^b^ | 1.16 | - | 2.03 |  | 1.53 | ^b^ | 1.15 | - | 2.05 |  | 1.09 |  | 0.80 | - | 1.48 |
|  |  | unchanged | 279/659 | 2.05 | ^c^ | 1.75 | - | 2.41 |  | 1.88 | ^c^ | 1.60 | - | 2.21 |  | 1.82 | ^c^ | 1.54 | - | 2.15 |  | 1.29 | ^b^ | 1.08 | - | 1.54 |
|  |  | decreased | 198/333 | 4.10 | ^c^ | 3.28 | - | 5.13 |  | 3.94 | ^c^ | 3.14 | - | 4.94 |  | 3.40 | ^c^ | 2.68 | - | 4.29 |  | 1.93 | ^c^ | 1.51 | - | 2.47 |
|  | unchanged | increased | 250/770 | 1.35 | ^c^ | 1.15 | - | 1.57 |  | 1.16 |  | 0.99 | - | 1.36 |  | 1.15 |  | 0.97 | - | 1.35 |  | 1.12 |  | 0.94 | - | 1.33 |
|  |  | unchanged | 2990/11353 | Reference | | |  |  |  | Reference | | |  |  |  | Reference | | |  |  |  | Reference | | |  |  |
|  |  | decreased | 412/734 | 3.58 | ^c^ | 3.08 | - | 4.17 |  | 3.37 | ^c^ | 2.89 | - | 3.93 |  | 3.20 | ^c^ | 2.74 | - | 3.75 |  | 2.24 | ^c^ | 1.90 | - | 2.65 |
|  | decreased | increased | 109/345 | 1.29 | ^a^ | 1.03 | - | 1.63 |  | 1.14 |  | 0.90 | - | 1.44 |  | 1.08 |  | 0.85 | - | 1.38 |  | 1.06 |  | 0.82 | - | 1.37 |
|  |  | unchanged | 227/824 | 1.06 |  | 0.91 | - | 1.25 |  | 1.09 |  | 0.93 | - | 1.28 |  | 1.07 |  | 0.91 | - | 1.27 |  | 0.95 |  | 0.80 | - | 1.13 |
|  |  | decreased | 94/165 | 3.70 | ^c^ | 2.71 | - | 5.05 |  | 3.73 | ^c^ | 2.71 | - | 5.12 |  | 3.13 | ^c^ | 2.25 | - | 4.34 |  | 2.45 | ^c^ | 1.74 | - | 3.45 |
| Model 1: adjusted for sex and age group.  Model 2: Model 1 + adjusted for marital status, educational level, living with a child/children under 12 years old (yes/no), residential area, smoking, drinking frequency, exercise frequency, time for house chores (increased/unchanged/decreased), time with family (increased/unchanged/decreased), body mass index, and disease currently being treated (yes/no).  Model 3: Model 2 + adjusted for type of industry, number of employees, commuting time, teleworking preference, telework frequency, job control, social support, and job stress (increased/unchanged/decreased).  ^a^*p*<0.05.  ^b^*p*<0.01.  ^c^*p*<0.001. | | | | | | | | | | | | | | | | | | | | | | | | | | |
